# Heterologous Expression and Delivery of Biologically Active Exendin-4 by *Lactobacillus paracasei* L14

**DOI:** 10.1371/journal.pone.0165130

**Published:** 2016-10-20

**Authors:** Zhu Zeng, Rui Yu, Fanglei Zuo, Bo Zhang, Deju Peng, Huiqin Ma, Shangwu Chen

**Affiliations:** 1 Beijing Advanced Innovation Center for Food Nutrition and Human Health, College of Food Science and Nutritional Engineering, China Agricultural University, Beijing, P. R. China; 2 Key Laboratory of Functional Dairy, Department of Food Science and Engineering, College of Food Science and Nutritional Engineering, China Agricultural University, Beijing, P. R. China; 3 Yangling Zhongyang Joint Ranch Co. Ltd., Beiyang Breeding Area, Yangling Street Agency, Yangling District, Xi'an, P. R. China; 4 College of Agriculture and Biotechnology, China Agricultural University, Beijing, P. R. China; Helsingin Yliopisto, FINLAND

## Abstract

Exendin-4, a glucagon-like protein-1 (GLP-1) receptor agonist, is an excellent therapeutic peptide drug for type 2 diabetes due to longer lasting biological activity compared to GLP-1. This study explored the feasibility of using probiotic *Lactobacillus paracasei* as an oral vector for recombinant exendin-4 peptide delivery, an alternative to costly chemical synthesis and inconvenient administration by injection. *L*. *paracasei* transformed with a plasmid encoding the exendin-4 gene (*L*. *paracasei* L14/pMG76e-exendin-4) with a constitutive promotor was successfully constructed and showed efficient secretion of exendin-4. The secreted exendin-4 significantly enhanced insulin secretion of INS-1 β-cells, along with an increment in their proliferation and inhibition of their apoptosis, corresponding to the effect of GLP-1 on these cells. The transcription level of the pancreatic duodenal homeobox-1 gene (*PDX*-1), a key transcription factor for cellular insulin synthesis and secretion, was upregulated by the treatment with secreted exendin-4, paralleling the upregulation of insulin gene expression. Caco-2 cell monolayer permeability assay showed a 34-fold increase in the transport of exendin-4 delivered by *L*. *paracasei* vs. that of free exendin-4 (control), suggesting effective facilitation of exendin-4 transport across the intestinal barrier by this delivery system. This study demonstrates that the probiotic *Lactobacillus* can be engineered to secrete bioactive exendin-4 and facilitate its transport through the intestinal barrier, providing a novel strategy for oral exendin-4 delivery using this lactic acid bacterium.

## Introduction

Glucagon-like peptide-1 (GLP-1) is a potent incretin produced by L-cells of the distal ileum and colon in response to nutrient intake [1]. It has a variety of antidiabetic effects that are mediated through β-cells in pancreatic islets and peripheral tissues. These include not only the promotion of glucose-dependent insulin secretion and inhibition of glucagon secretion [2, 3], but also inhibition of food intake and body weight gain by delaying gastric emptying [3], increasing β-cell mass [4, 5], and enhancing insulin sensitivity [6, 7]. However, despite its favorable antidiabetic functions, the native form of GLP-1 is unsuitable for therapeutic use because of its extremely short half-life *in vivo* (~1–2 min), due to its rapid inactivation by the ubiquitous enzyme dipeptidyl peptidase IV (DPP-IV) [8]. Two GLP-1-based strategies have been adopted to solve this problem: DPP-IV-resistant GLP-1 analogs and DPP-IV inhibitors [9]. Sitagliptin, saxagliptin and vildagliptin are examples of currently available DPP-IV inhibitors. Their major advantage is that they can be administered orally; however, many other hormones are also substrates of DPP-IV, and DPP-IV inhibition may affect the pathways mediated by those hormones [10].

Exendin-4, the first discovered GLP-1 mimetic (with 53% homology), was originally isolated from the salivary secretions of the Gila monster *Heloderma suspectum* [11]. Exendin-4 binds to and activates the GLP-1 receptor, and thus shares many antidiabetic actions with GLP-1, such as glucose-dependent increase of insulin secretion, suppression of glucagon secretion, reduction of food intake and facilitation of β-cell neogenesis [12–15]. Exendin-4 has a longer biological half-life than GLP-1 due to its resistance to DPP-IV degradation, and can serve as a potent and longer-acting agonist of the pancreatic GLP-1 receptor [16]. Synthetic exendin-4 (exenatide) was approved as the first GLP-1 receptor agonist for diabetes treatment by the US Food and Drug Administration (FDA) in 2005. Today, an exenatide extended-release suspension (Bydureon) is available, administered by weekly injection [17].

Oral administration is the preferred method of drug delivery due to patient acceptance, long-term compliance and few side effects [18, 19]. However, this strategy is usually not feasible for protein or peptide drugs due to poor bioactivity resulting from proteolysis during passage though the gastrointestinal environment and poor epithelial permeability [20]. Many approaches have been explored to increase the oral bioavailability of antidiabetic peptides by protecting them from enzymatic digestion and increasing their absorption across the intestinal wall [19, 20]. Some of the most widely studied strategies include the use of nanoparticles [20, 21], mucoadhesive polymers [22], absorption enhancers [23] and oral films [24]. Although these strategies have provided novel prospects for the problem, most of them have encountered roadblocks such as low efficacy and high toxicity, and have barely advanced to clinical trials [25].

In the past two decades, lactic acid bacteria (LAB) have been demonstrated as promising oral delivery vehicles for protein/peptide drugs [26–28]. Before that, LAB were used for centuries in traditional fermented products in the food industry; they are generally regarded as safe (GRAS) by the FDA. In addition, specific strains of LAB, and especially of *Lactobacillus*, have many intrinsic probiotic effects on human and animal health, such as maintaining gastrointestinal homeostasis and modulating immunity [29]. Furthermore, many *Lactobacillus* strains can survive transit through the human gastrointestinal tract and tightly adhere to the intestinal epithelial cells, facilitating mucosal targeting and transport of therapeutic molecules while minimizing bacterial and enzymatic degradation of the protein-based drugs [30, 31]. Because of these advantages, LAB have been regarded as an appealing potential alternative to other oral delivery systems. A study on the use of interleukin (IL)-10-transformed *Lactococcus lactis* to treat Crohn’s disease has passed phase I clinical trials [32]. Several articles exploring the potential of transformed LAB for diabetes treatments have also been published, including the use of *L*. *lactis* to deliver insulin analog [33–35] or combined expression of IL-10 and glutamic acid decarboxylase (GAD65) [36], and the use of *Bifidobacterium longum* to deliver GLP-1 [37] or *Lactobacillus gasseri* to deliver GLP-1(1–37) [38]. Here, we evaluated whether *Lactobacillus paracasei* can secrete bioactive exendin-4, to provide an alternative route for the use of LAB as a vector for oral delivery of exendin-4.

In this study, a *L*. *paracasei* strain transformed for expression and secretion of bioactive exendin-4 was explored as a novel delivery system for this GLP-1 mimetic. The expression characteristics of exendin-4 in *L*. *paracasei* were assayed and the bioactivities of recombinant exendin-4 were evaluated on the *in vitro* pancreatic β-cell line INS-1 as a model. The transport of exendin-4 through intestinal epithelial cells was also assayed using Caco-2 cell monolayers *in vitro*.

## Materials and Methods

### Materials

The Caco-2 cell line and the rat insulinoma cell line INS-1were purchased from American Type Culture Collection. Dulbecco’s modified eagle medium (DMEM), Roswell Park Memorial Institute medium (RPMI) 1640, fetal bovine serum (FBS), non-essential amino acids (100X), and 0.25% trypsin with EDTA were obtained from Gibco BRL Life Technology. Transwell^®^ inserts, treated tissue culture (0.4 μm pore size, 4.7 cm^2^ surface area) and culture flasks were purchased from Costar Corporation (Corning, NY).

### Bacterial strains, plasmids, and growth conditions

The bacterial strains and plasmids used in this study are listed in Table 1. *Escherichia coli* strains were grown at 37°C in Luria-Bertani (LB) medium with shaking at 200 rpm. *L*. *paracasei* L14 was aerobically propagated statically at 37°C in de Man, Rogosa and Sharpe (MRS) medium. Antibiotics were used at the following concentrations: 500 μg/ml erythromycin or 100 μg/ml ampicillin for *E*. *coli* and 5 μg/ml erythromycin for lactobacilli.

**Table 1:** Bacterial strains and plasmids used in this study.

**Strains/plasmids**	**Description**	**Source or reference**
**Strains**		
*Lactobacillus paracasei* L14	Wild-type, isolated from milk	CGMCC NO. 4015
***L*. *paracasei* L14/pMG76e**	*L*. *paracasei* L14 harboring empty vector pMG76e	This study
***L*. *paracasei* L14/pMG76e-exendin-4**	*L*. *paracasei* L14 harboring pMG76e-exendin-4	This study
**Plasmids**		
pMG76e	Derivative of pMG36e, 3.5-kb, Emr, containing the P32 promoter	Laboratory stock
pMG76e-**exendin-4**	pMG76e carrying *exendin-4* gene	This study

### DNA manipulation and transformation

The *exendin-4* cDNA sequence was obtained from GenBank (DI206426.1) and was codon optimized for LAB using the Codon Adaptation Tool (JCAT). An oligonucleotide carrying a fusion of signal peptide Usp45 and LEISSTCDA propeptide sequences, *exendin-4*, 6×His tag and *Xba* I and *Xho* I cDNA restriction sites between Usp45 and the 6×His tag was synthesized by GENEWIZ Biological Technology Co. Ltd. (Suzhou, China). This was incorporated into pMG76e vector (derived from pMG36e [39]) to yield pMG76e-exendin-4. The pMG76e-exendin-4 and empty control pMG76e plasmids were transformed into competent *L*. *paracasei* L14 cells by electroporation.

Heterologous expression and secretion of exendin-4 by *L*. *paracasei* L14

To detect exendin-4 expression, the recombinant *L*. *paracasei* L14/pMG76e-exendin-4 and control (*L*. *paracasei* L14/pMG76e) transformants were grown overnight in MRS medium supplemented with 5 μg/ml erythromycin. A 1% (v/v) inoculum of the *L*. *paracasei* transformants was transferred into 50 ml fresh MRS medium and incubated statically at 37°C for 12 h; 100 μl of culture supernatant was sampled every 2 h for ELISA assay of exendin-4 secretion.

To prepare exendin-4-conditioned medium for INS-1 cell experiments, RPMI 1640 medium was used for exendin-4 secretion (RPMI 1640–exendin-4 medium). Overnight cultures of *L*. *paracasei* L14/pMG76e-exendin-4 and *L*. *paracasei* L14/pMG76e were diluted 1:100 in fresh MRS medium and incubated at 37°C for 12 h, to a bacterial cell density of about 10^9^ cfu/ml. Cells were then harvested and washed twice in PBS (pH 7.4) buffer, suspended in RPMI 1640 buffered with 20 mM HEPES (pH 7.4), and incubated for 12 h. The culture was centrifuged at 8000 × *g* for 10 min at 4°C. The supernatant was collected, adjusted to pH 7.4 with sodium hydroxide, and sterilized by filtration through a 0.22-μm membrane as RPMI 1640–exendin-4 medium.

### Recombinant exendin-4 extraction and western blot analysis

The transformed *L*. *paracasei* L14 strain cultures (10 ml of 10^9^ or 10^10^ cfu/ml cells) were centrifuged at 6000 × *g* for 10 min at 4°C. Soluble cytoplasmic and secreted protein fractions were respectively prepared. The cell pellets were washed three times with PBS, resuspended in 1 ml PBS buffer (pH 7.4) and ultrasonically disrupted. Cell debris were separated out by centrifugation and discarded. The supernatant was subjected to precipitation with trichloroacetic acid (10% w/v) for 2 h at 4°C; then the mixture was centrifuged at 12,000 × *g* for 10 min, and the pellet was washed three times with ice-cold acetone and then dissolved in 50 μl PBS. Both cytoplasm and medium extract samples were mixed with 4× SDS loading buffer (12% w/v SDS, 6% v/v β-mercaptoethanol, 30% w/v glycerol, 0.05% w/v Coomassie blue G-250, 150 mM Tris-HCl pH 7.0), and subjected to Tricine SDS-PAGE (16% T, 6% C) analysis as described previously [40]. Proteins on the polyacrylamide gel were then semi-dry-electrotransferred to a PVDF membrane (Millipore). The 6×His-tagged exendin-4 proteins were analyzed by Western blotting with 6×His antibody (Cell Signaling Technology), horseradish peroxidase-conjugated goat anti-rabbit polyclonal IgG secondary antibody (Cell Signaling Technology) and the ECL Chemiluminiscent Substrate (Pierce) according to manufacturer's instructions.

### Indirect ELISA quantification of secreted exendin-4

The concentration of exendin-4 secreted by recombinant *L*. *paracasei* was measured by indirect ELISA according to the method described by Zhang et al. [41] with some modifications. Standard exendin-4 (Sigma, 0.2 μg/ml) was used as the coated antigen. Equal volumes (100 μl) of monoclonal antibody against exendin-4 (Abcam) (1:4000) and standard exendin-4 (0–100 nmol/l) or culture supernatant samples of *L*. *paracasei* L14/pMG76e-exendin-4, preincubated overnight at 4°C, were applied to detect the captured exendin-4. Horseradish peroxidase-conjugated rabbit anti-mouse IgG (Abcam) (100 μl) was used at a 1:10,000 dilution and revealed by reaction with TMBS substrate (Amresco). Absorption was determined at 450 nm and 630 nm by Multiskan MK3 ELISA plate reader (Thermo Labsystems). The exendin-4 concentration was calculated by the standard curve method.

### Cell culture of rat insulinoma cell line INS-1

INS-1 cells (passage 10–20) were cultured in RPMI 1640 medium containing 10% (v/v) FBS, 1 mM sodium pyruvate, 50 μM β-mercaptoethanol, 10 mM HEPES, 100 U/ml penicillin and 100 μg/ml streptomycin, and cells were maintained at 37°C in a humidified (5% CO_2_, 95% air) atmosphere.

### Insulin secretion assay

INS-1 cells were seeded in 24-well plates at a density of 10^5^ cells per well and grown to 100% confluence before the assay. The medium was changed 24 h before the assay. For the assay, cells were quickly rinsed in 1 ml Hank's balanced salt solution (HBSS) [42] with 3 mM glucose followed by a 2 h preincubation with 1 ml of the same buffer. Then the buffer was discarded, RPMI 1640–exendin-4 medium diluted 1:1 (v/v) with fresh medium plus 5 mM glucose (600 μl per well) were added, and the mixture was incubated for 2 h. Standard GLP-1 (100 nmol/l) in RPMI 1640 medium was used as a positive control. Preliminary experiments indicated that 5 mM glucose maximizes glucose-dependent insulin secretion of INS-1 cells stimulated by GLP-1 (S1 Fig). At the end of the incubation, supernatant was collected and centrifuged for 10 min at 4000 × *g* at 4°C and insulin concentration was determined by ELISA kit (Millipore, St. Charles, MO, USA). The cells were collected to analyze mRNA expression of pancreatic duodenal homeobox-1 (*PDX*-1) and insulin gene (*Insulin*).

### Quantitative reverse transcription PCR (qRT-PCR) analysis of expression of *PDX*-1 and *Insulin*

Total RNA was isolated from INS-1 cells using TRIzol Reagent (Invitrogen). Reverse transcription was performed using PrimeScript II first-strand cDNA synthesis kit (Takara, China), containing 1 μg of total RNA as the template. qRT-PCR was carried out using a SYBR Green assay kit (Tiangen). Specific primers for *PDX*-1, *Insulin* and the internal control gene β-actin (*Actb*) from *Rattus norvegicus* were designed with Primer Premier 5 software (Table 2). Gene expression was normalized by the ΔΔC_T_ method [43] using β-actin as the reference for the calculations. The experiment was carried out in triplicate and the average results are reported.

**Table 2:** Primers used in this study.

**Name**	**Sequence (5 ′→3′)**	**NCBI Gene ID of** *Rattus norvegicus*
β-actin-F	CCACCCGCGAGTACAACC	*Actb*, Gene ID: 81822
β-actin-R	CCACGATGGAGGGGAAGAC	
PDX-1-F	GGTGCCAGAGTTCAGTGCTAA	*Pdx*-1, Gene ID: 18609
PDX-1-R	CCAGTCTCGGTTCCATTCG	
insulin-F	CCTGCCCAGGCTTTTGTCAA	Degenerate primers of *Insulin* (*Ins*1 and *Ins*2), Gene ID: 24505 & 24506, respectively
insulin-R	CCTCCAGTGCCAAGGTCTGAAG	

### Cell-proliferation and apoptosis assay

INS-1 cells were seeded in 96-well plates at a density of 2 × 10^4^ cells per well. After 36 h incubation, the cells were grown to nearly 60% confluence and the medium was replaced with RPMI 1640–exendin-4 medium diluted 1:1 (v/v) with fresh RPMI 1640 containing 10% FBS (100 μl per well). Standard GLP-1 in RPMI 1640 medium (100 nmol/l) was used as a positive control and pure RPMI 1640 medium was used as a blank control. The mixture was incubated for 24 h. The PI3K (phosphatidylinositol-3-kinase) antagonist treatment cells were preincubated with wortmannin (100 nmol/l) for 15 min before the treatment. The number of viable cells was then analyzed by cell counting kit-8 (CCK-8) assay (Biyuntian Biotech Co. Ltd.) based on the formation of formazan with sensitive changes in OD_450_ values. Briefly, at the end of the 24-h incubation, the medium was discarded and cells were washed twice in RPMI 1640 and incubated in a 100-μl mixture (with 90 μl RPMI 1640 and 10 μl CCK-8) in each well for 4 h. The absorbance was read at 450 nm using a Multiskan MK3 ELISA plate reader to determine the *in vitro* cell proliferation. For apoptosis assays, staurosporine (1 μmol/l) was added in all treatment groups for 1 h, after which the cells were treated with Annexin V-PI (propidium iodide) cell apoptosis kit (Biyuntian Biotech Co. Ltd.) and apoptosis was measured by flow cytometry using a FACScan (Becton Dickinson).

### Transport of exendin-4 through Caco-2 monolayer

Caco-2 cells (10–20 passages) were cultivated in 25-cm^2^ flasks in DMEM supplemented with 10% (v/v) FBS, 1% (v/v) non-essential amino acids and 100 IU/ml penicillin–100μg/ml streptomycin (s-DMEM) at 37°C in an atmosphere of 5% CO_2_ and high humidity. Upon 80% confluence, cells were harvested using 0.25% trypsin EDTA and seeded on the polyester inserts (0.4 μm pore size, 24 mm diameter, 4.67 cm^2^ grown surface, Costar, Corning, NY) in 6-well transwell plates at a density of 10^5^ cell/cm^2^ and incubated under normal cell culture conditions. The culture medium was replaced every other day for the first week and then every day until it was ready for transport studies when the monolayer became confluent and the transepithelial electrical resistance (TEER) reading reached a plateau (about 21 days). The TEER was monitored according to the method described by Hubatsch et al [44].

After the Caco-2 monolayer cell became confluent, the culture medium was washed twice by PBS (pH 7.4). Then 1.5 ml of freshly made L14/pMG76e-exendin-4 cells in antibiotic-free s-DMEM (~10^8^ cfu/ml) was added to the apical side and 2.6 ml of fresh s-DMEM without antibiotics was added to the basolateral side. Caco-2 cell viability was then measured by trypan blue assay at 0, 2, 4 and 6 h after the addition of L14/pMG76e-exendin-4 cells. To assay exendin-4 transport through the Caco-2 monolayer, the plate was centrifuged at 45 × *g* for 5 min after the bacterial cells were added and incubated under normal culture conditions. At predetermined time points, 100 and 200 μl supernatant samples were withdrawn from the apical and basolateral sides, respectively, for exendin-4 quantification by ELISA and replaced with an equal volume of s-DMEM without the antibiotics. The normalized transport rate of exendin-4 was calculated according to Agarwal et al. [35], and TEER was monitored as well.

### Statistical analysis

All experiments were performed in triplicate, and data were expressed as mean ± SD. All analyses were carried out by one-way ANOVA and significant differences (*P* < 0.05) between means were identified by Duncan’s test using SPSS 20 (SPSS Inc.).

## Results

### Expression of recombinant exendin-4 in *L*. *paracasei* L14

**Fig 1:**
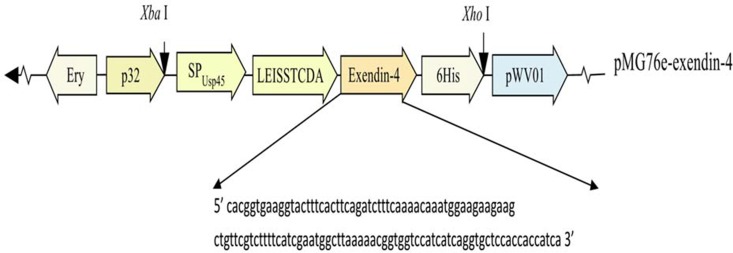
Expression cassette for recombinant exendin-4 secretion by *L*. *paracasei* strain L14. Expression of the recombinant exendin-4 was controlled by the constitutive p32 promoter. SP_Usp45_: sequence encoding the signal peptide Usp45; LEISSTCDA: sequence encoding the synthetic LEISSTCDA propeptide; the codon-optimized cDNA sequence of exendin-4 is also supplied.

**Fig 2:**
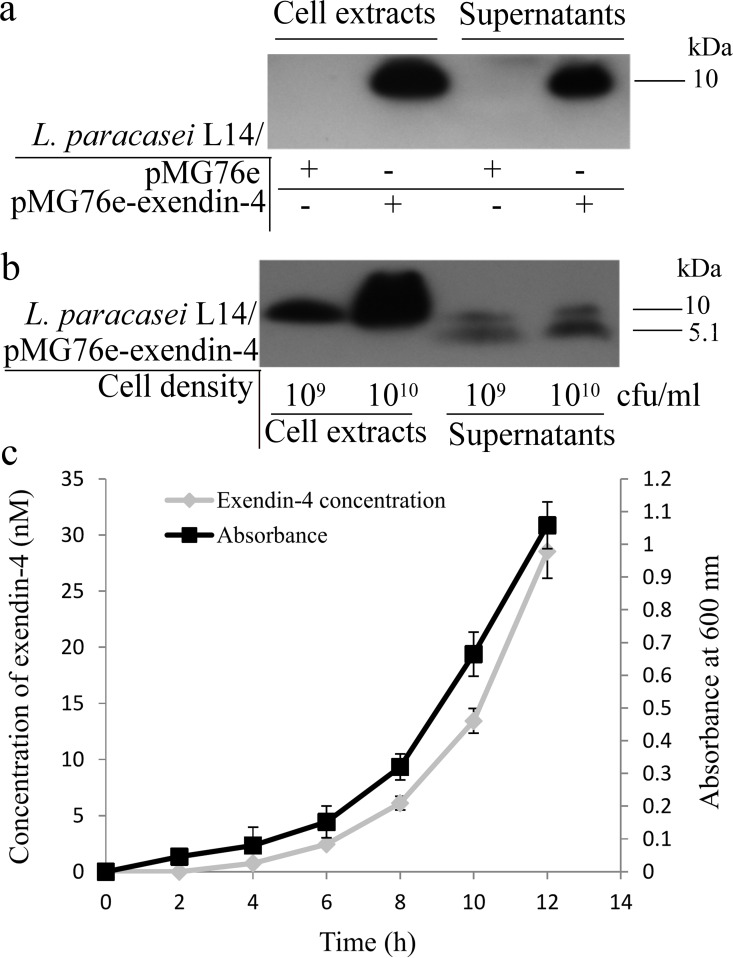
Western blotting and enzyme-linked immunosorbent assay (ELISA) analysis of recombinant exendin-4 produced by plasmid-transformed *L*. *paracasei* L14 strains. (a) Strains cultured in MRS medium: lanes 1 and 2, cell pellet of *L*. *paracasei* L14/pMG76e and *L*. *paracasei* L14/pMG76e-exendin-4 strains, respectively; lanes 3 and 4, excretion of *L*. *paracasei* L14/pMG76e and *L*. *paracasei* L14/pMG76e-exendin-4 strains, respectively. (b) Strains cultured in buffered RPMI 1640 medium: lanes 1 and 2, cell pellet extraction of *L*. *paracasei* L14/pMG76e-exendin-4 strain with bacterial concentration of 10^9^ cfu/ml and 10^10^ cfu/ml, respectively; lanes 3 and 4, cell excretion of *L*. *paracasei* L14/pMG76e-exendin-4 strain with bacterial concentration of 10^9^ cfu/ml and 10^10^ cfu/ml, respectively. (c) ELISA of exendin-4 expression *in vitro* at different time points of culture as described in the methods (three repeat expressions, means ± SD).

Fig 1 shows the plasmid pMG76e-exendin-4 and the cDNA sequence of the codon-optimized exendin-4. The constructed plasmids pMG76e and pMG76e-exendin-4 were introduced into *L*. *paracasei* L14 and the resultant transformant culture and cells were prepared for the assays. Soluble cytoplasmic and secreted protein fractions were extracted from cells and culture media and subjected to Tricine SDS-PAGE and immunoblotting assays (Fig 2). Fig 2A shows the western blot analysis of recombinant exendin-4 of transformed strains cultured in MRS medium. A prominent 9.4-kDa band can be seen in the supernatants of both the bacterial culture and the soluble cytoplasmic proteins from recombinant strain *L*. *paracasei* L14/pMG76e-exendin-4. This band matched the calculated molecular weight corresponding to the primary protein, containing the signal peptide Usp45 of the recombinant exendin-4. Thus, the signal peptide in the MRS supernatant could not be cleaved by *L*. *paracasei* signal peptidase.

RPMI 1640 medium buffered with 20 mM HEPES (pH 7.4) was conditioned with the transformant strain of *L*. *paracasei* L14/pMG76e-exendin-4 and used at different bacterial cell concentrations (10^9^ cfu/ml and 10^10^ cfu/ml) to secrete exendin-4. The secreted exendin-4 showed multiple proteoforms (Fig 2B): a preprotein of 9.4 kDa was found in the soluble cytoplasmic fraction, and two protein bands, the 9.4-kDa preprotein and the mature 5.1-kDa protein with successful cleavage of the signal peptide, appeared in supernatants of the transformed strain. The bacteria at 10^10^ cfu/ml produced more recombinant exendin-4 than bacteria at 10^9^ cfu/ml, indicating that exendin-4 expression and secretion levels are positively related to bacterial density. Prolonged incubation of the transformed *L*. *paracasei* strain in buffered RPMI 1640 medium produced mature exendin-4, implying that buffered RPMI 1640 medium may provide a more suitable biochemical environment for correct sorting and processing of secreted exendin-4 by the bacterial signal peptidases.

Fig 2C shows the growth curve of the transformed strain, *L*. *paracasei* L14/pMG76e-exendin-4, and its secretion of exendin-4. The secretion of exendin-4 by *L*. *paracasei* L14/pMG76e-exendin-4 was consistent with the bacterial growth profile. Total secretion of exendin-4 reached 28.5 nmol/l in 50 ml culture volume after 12 h incubation.

### Recombinant exendin-4's insulinotropic effect in INS-1 cells

**Fig 3:**
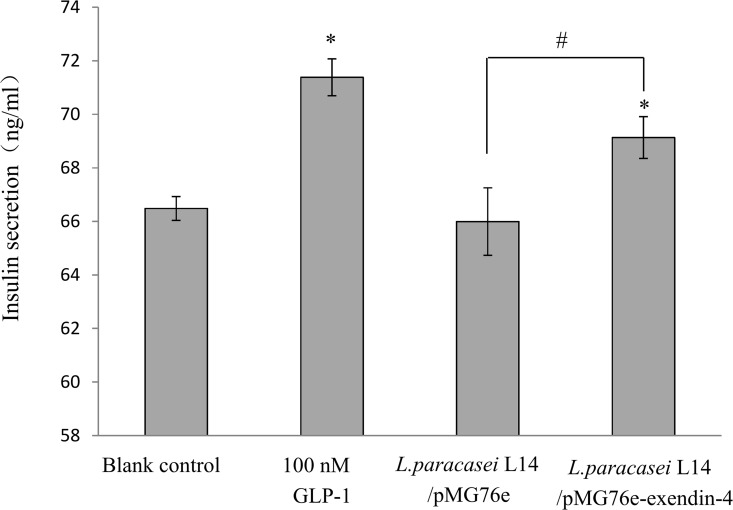
Effect of recombinant exendin-4 on insulin secretion of INS-1 cells (means ± SD, n = 3). *p < 0.05 vs. control cells with pure cell culture media; ^##^*p* < 0.01 vs. *L*. *paracasei* L14/pMG76e-treated group.

To determine whether the exendin-4 protein secreted by *L*. *paracasei* L14/pMG76e-exendin-4 retains its biological activity, its ability to stimulate insulin secretion was evaluated with rat pancreatic β-cell line INS-1. As observed in Fig 3, *L*. *paracasei* L14/pMG76e–exendin-4 medium significantly stimulated insulin secretion to 69.1 ± 0.3 ng/ml, which was 4% higher than the blank control (with only RPMI 1640, 66.5 ± 0.5 ng/ml, added) (*p* < 0.05), and 5% higher than the negative control (*L*. *paracasei* L14 with empty plasmid pMG76e-conditioned medium, 65.9 ± 0.8 ng/ml) (*p* < 0.05). Similarly prepared medium from *L*. *paracasei* L14 transformed with empty plasmid pMG76e failed to increase insulin secretion. The GLP-1 standard solution also displayed insulinotropic ability, which enhanced insulin secretion to 71.4 ± 0.7 ng/ml, 7% higher than the blank control (*p* < 0.05). Thus, it could be concluded that the *Lactobacillus*-secreted exendin-4 is bioactive and has an insulinotropic effect.

### Recombinant exendin-4 increases mRNA expression of *PDX-1* and the *insulin* gene

**Fig 4:**
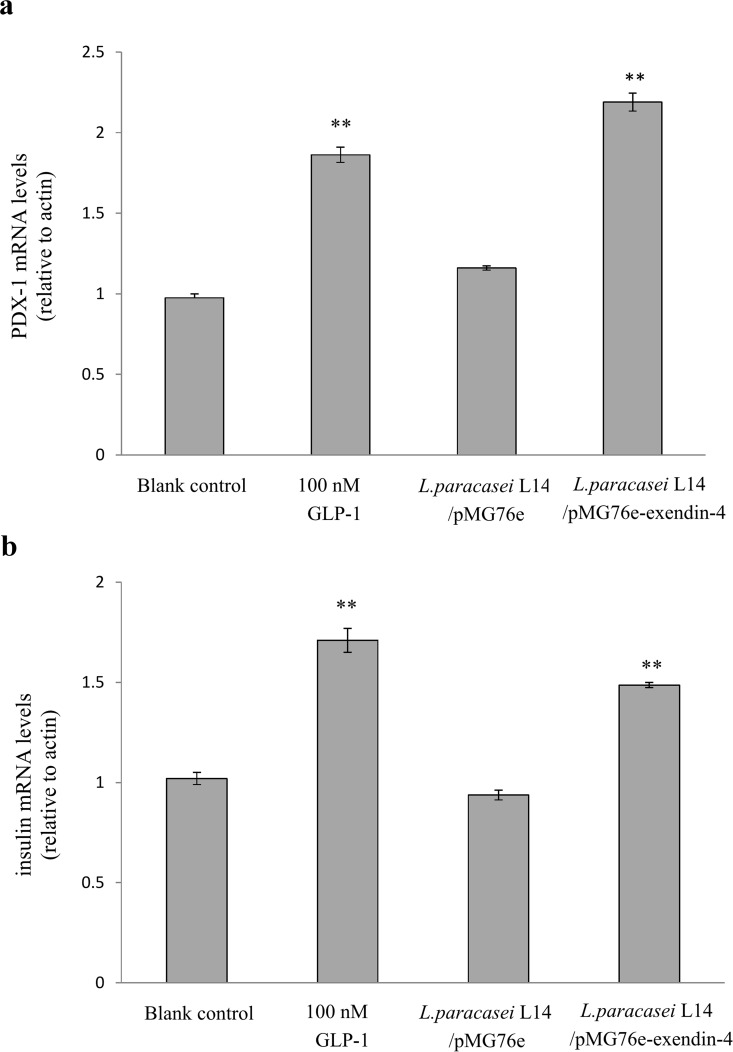
Effect of recombinant exendin-4 on the transcription of *PDX-1* and *insulin* gene in INS-1 cells. (a) *PDX*-1 mRNA expression level (means ± SD, n = 3). (b) *Insulin* mRNA expression level (means ± SD, n = 3). **p < 0.05 vs. control cells with pure cell culture media and *L*. *paracasei* L14/pMG76e-treated group.

PDX-1 is an important transcription factor, which plays an essential role in GLP-1-enhanced insulin transcription and synthesis, pancreas development and β-cell neogenesis [45–47]. GLP-1 can increase pancreatic expression of PDX-1, enhancing the transcription and synthesis of insulin [47]. Thus, we also investigated mRNA expression levels of *PDX-1* and the *insulin* gene in INS-1 cells treated with RPMI 1640–exendin-4 medium for 2 h. As shown in Fig 4A, *L*. *paracasei* L14/pMG76e–exendin-4 medium significantly increased *PDX-1* mRNA expression (*p* < 0.01), which was 2.2-fold higher than in the blank control and 1.8-fold higher than in *L*. *paracasei* L14/pMG76e medium. The GLP-1 standard solution also increased *PDX-1* mRNA expression to 1.9-fold that of the blank control. *Insulin* mRNA expression was consistent with that of *PDX-1* (Fig 4B). *L*. *paracasei* L14/pMG76e–exendin-4 medium increased *insulin* mRNA expression 1.5-fold (*p* < 0.01) and 1.6-fold (*p* < 0.01) that of the blank control and *L*. *paracasei* L14 harboring empty plasmid, respectively. The standard GLP-1 also promoted *insulin* gene transcription (*p* < 0.01), which was 1.7-fold higher than the blank control. Taken together, these results suggested that the expression of *PDX-1* is upregulated by *L*. *paracasei* L14-secreted exendin-4, thereby exerting the functional effects of enhancing insulin transcription and synthesis.

### Recombinant exendin-4 enhances INS-1 cell proliferation and decreases staurosporine-induced cell apoptosis

**Fig 5:**
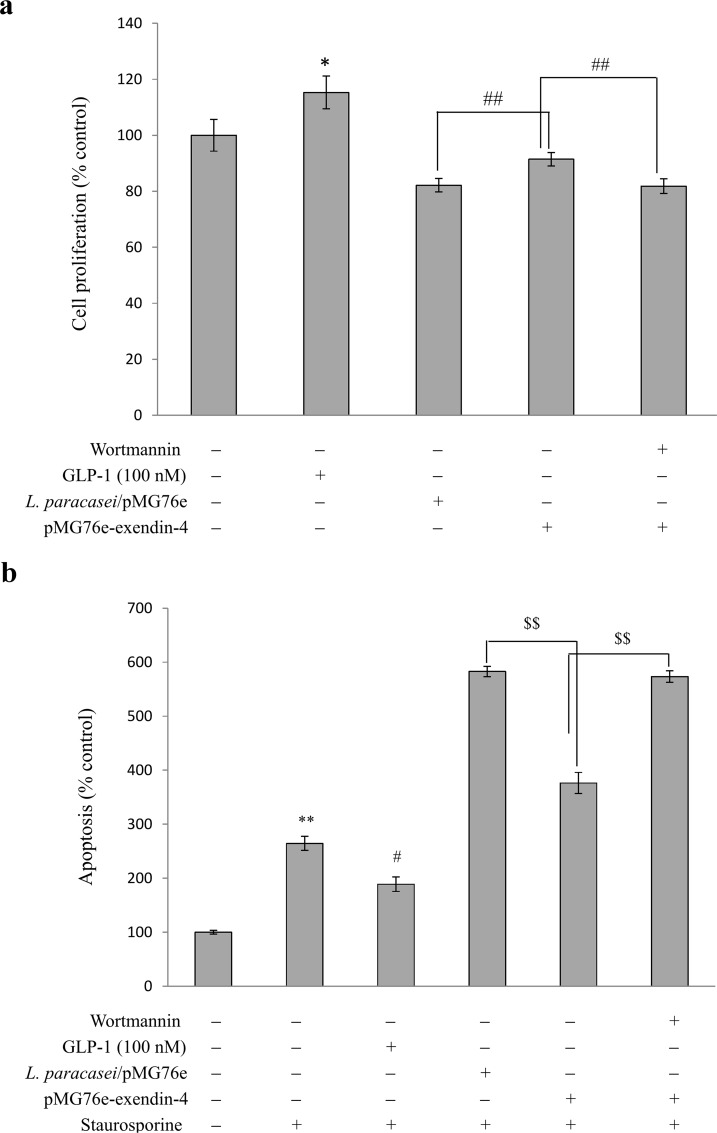
Effect of recombinant exendin-4 on proliferation and staurosporine-induced apoptosis of INS-1 cells. (a) INS-1 cells were preincubated with media alone or with 100 nmol/l wortmannin, followed by treatment with different agents as indicated for 18 h (means ± SD, n = 3). *p < 0.05 vs. control cells with pure cell media; ##*p <* 0.01. (b) INS-1 cells were preincubated with media alone or with 100 nmol/l wortmannin, followed by treatment with different agents as indicated for 18 h. Then cells were treated with media or 1 mmol/l staurosporine, as indicated (means ± SD, n = 3). **p < 0.01 vs. control cells with no staurosporine; #*p <* 0.05 vs. cells with only staurosporine; ^$ $^*p <* 0.01.

GLP-1, and its long-acting analog exendin-4, can also increase proliferation and decrease apoptosis of β-cells [4, 48]; both of these effects are prevented by treatment with wortmannin, a specific pharmacological inhibitor of PI3K [4]. Therefore, we determined whether the exendin-4 secreted by *L*. *paracasei* is also functionally involved in regulating proliferation and apoptosis of β-cells using the INS-1 cell model. As shown in Fig 5A, both conditioned media—with pMG76e and pMG76e-exendin-4 transformant culture—inhibited INS-1 cell growth compared to the blank control group. However, compared to pMG76e, pMG76e-exendin-4 transformant-conditioned medium significantly increased cell proliferation (*p* < 0.01), by 13.5%. On the other hand, when wortmannin was added, there was no significant difference in cell proliferation between the pMG76e and pMG76e-exendin-4 treatments, indicating that the recombinant exendin-4 improves INS-1 cell proliferation via the PI3K signal pathway. The positive control, 100 nmol/l standard GLP-1, also increased cell proliferation (*p* < 0.05), which was 15.3% higher than with the blank control.

After a 1 h treatment with staurosporine, a general kinase inhibitor which induces a broad spectrum of cell apoptosis [49, 50], the number of earliest-stage apoptotic cells was significantly increased to 264.3% of the control with no staurosporine (*p <*0.01); pretreatment with standard GLP-1 reduced the apoptosis to 170% (*p* < 0.05) (Fig 5B). The number of earliest-stage apoptotic cells was 582% of the control upon pretreatment with pMG76e culture, while the number was significantly reduced to 376% (*p <* 0.01) when pretreatment was with pMG76e-exendin-4 culture. When wortmannin was added, apoptotic cell number following pMG76e-exendin-4 treatment was 562%, not significantly different from the pMG76e treatment. These results showed that the exendin-4 secreted by *L*. *paracasei* L14 decreases staurosporine-induced apoptosis via the PI3K signal pathway.

### Delivery of exendin-4 by L14/pMG76e-exendin-4 through Caco-2 cell monolayer

**Fig 6:**
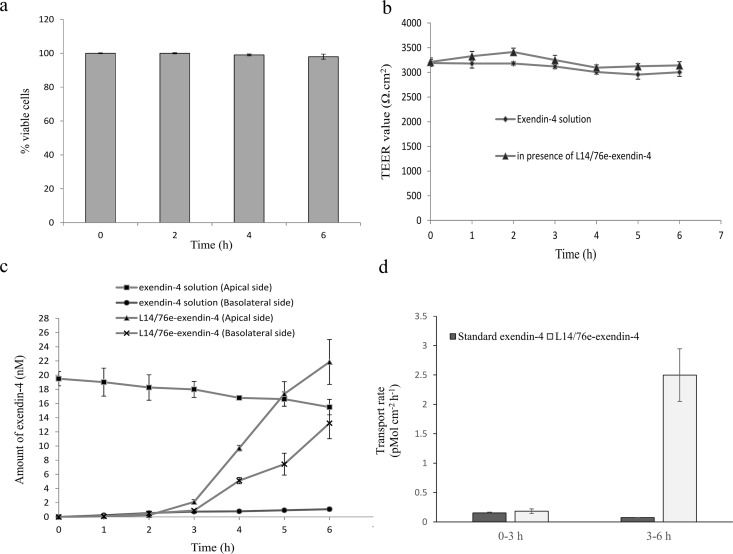
Transport of exendin-4 through Caco-2 cell monolayers. (a) Caco2 cell viability when co-cultured with strain L14/pMG76e-exendin-4. (b) TEER values of the Caco-2 cell monolayer in the presence of L14/pMG76e-exendin-4 or standard exendin-4 solution (control) during the exendin-4 transport study (means ± SD, n = 3). L14/pMG76e-exendin-4 suspension in s-DMEM or standard exendin-4 in s-DMEM was added to the apical side of all wells and incubated for 6 h. (c) Transport of exendin-4 across the Caco-2 monolayers (means ± SD, n = 3). Samples from both the apical and basolateral side of the transwell were taken for the assay of exendin-4 concentration by ELISA (means ± SD, n = 3). (d) Normalized transport rate of exendin-4 through Caco-2 monolayers (means ± SD, n = 3).

The effect of L14/pMG76e-exendin-4 on cell viability in the Caco-2 monolayer was analyzed by the trypan blue method. As shown in Fig 6A, cell viability was 100, 99 and 98% at 2, 4 and 6 h compared to no treatment, respectively. Differences in Caco-2 cell viability in the absence vs. presence of L14/pMG76e-exendin-4 were not significant (*p >*0.05).

TEER was monitored to explore the integrity of the Caco-2 cell monolayer throughout the experimental period. As shown in Fig 6B, during the transport studies, the TEER values were nearly stable and there was no statistical difference between the wells in the presence or absence of L14/pMG76e-exendin-4, indicating that the integrity of the cell monolayer was not compromised.

Fig 6C shows the absorption of exendin-4 through the Caco-2 monolayer when delivered by the transformed strain L14/pMG76e-exendin-4 or standard exendin-4 solution (control). During the first 3 h, the exendin-4 transport rates through the cell monolayer was 0.18 ± 0.002 and 0.15 ± 0.013 pMol cm^-2^ h^-1^ for L14/pMG76e-exendin-4 and the standard exendin-4, respectively. However, from 3–6 h, the transport rate of L14/pMG76e-exendin-4-delivered exendin-4 reached 2.5 ± 0.45 pMol cm^-2^ h^-1^ (Fig 6D), which was 34 times that of the exendin-4 control, indicating that the living *L*. *paracasei* delivery system has a much higher efficiency for exendin-4 absorption and transportation. This high delivery efficiency is probably due to the bacteria's strong adherence to the monolayer surface leading to an increased concentration of exendin-4 close to the epithelial cell monolayer [31, 51].

## Discussion

Exendin-4, a long-acting agonist of the GLP-1 receptor, shares many antidiabetic actions with GLP-1, and has become an important therapeutic drug for diabetes. However, subcutaneous injection is still the common administration route for such peptide drugs.

In this study, we developed a novel oral delivery system for exendin-4 by using an engineered *Lactobacillus* strain as the carrier. Lactobacilli, as the major inhabitants of the human intestinal tract, can reach and colonize the intestine and play an important role in maintaining a healthy microbiota, as well as contributing to many beneficial functions for human health [29]. These properties make lactobacilli better candidates as live oral vectors for mucosal delivery of prophylactic and therapeutic molecules than *Lactococcus lactis*. We developed recombinant *L*. *paracasei* as an oral vector for the live delivery of exendin-4 *in situ*, taking advantage of its probiotic colonization of the intestinal tract.

To guarantee an engineered strain of *L*. *paracasei* L14 with considerable exendin-4 secretion, the Usp45 signal peptide and LEISSTCDA were concatenated as the signal and leading peptides of recombinant exendin-4 [52] (Fig 1). Western blot confirmed expression of the exendin-4 proprotein in both supernatant and cells of the bacteria (Fig 2A); cleavage of the recombinant signal peptide, yielding mature exendin-4 with the expected molecular mass of 5.1 kDa, was obtained from the proteoforms of exendin-4 under the proper pH conditions (Fig 2B), due to the putative influence of medium pH on the processing of the secreted recombinant protein. When grown in MRS medium for 12 h, *L*. *paracasei* L14 acidifies the growth medium to pH 4.0–4.6, whereas when it is grown in buffered RPMI 1640 medium for 12 h, the pH value is maintained at 6.0–7.4. Previous studies have suggested that culture pH is an important factor in regulating secreted recombinant protein for correct sorting, processing, and maintenance of stability and bioactivity. It was further revealed that an acid environment decreases the stability and bioactivity of secreted proteins [33, 53, 54,]. Thus, we hypothesize that the mature exendin-4 develops in buffered RPMI 1640 medium primarily because its near-neutral pH leads to better protein processing. In addition, studies have reported that lactobacilli reside mainly in the human ileum, jejunum, and colon [55, 56], which have pH values of about 6.0–8.0 [57]. These values are close to that of the buffered RPMI 1640 medium in which the mature exendin-4 developed.

Quantification assay showed secretion of 28.5 nmol/l exendin-4 after 12 h (Fig 2C). When co-cultured with Caco-2 cells, total secretion of exendin-4 reached 70.4 pMol/10^8^ cfu within 6 h following a linear increment, and about 51% of this was absorbed and transported by the Caco-2 cell monolayer (Fig 6C). The secreted and processed exendin-4 protein had an insulinotropic effect on the rat pancreatic islet β-cell line INS-1, comparable to the positive control (100 nmol/l GLP-1) (Fig 3). The GLP-1 analog exendin-4 is known to have important antidiabetic effects, mainly through promotion of insulin secretion and insulin synthesis via upregulation of *PDX*-1 gene expression [47, 58], facilitation of β-cell neogenesis and inhibition of β-cell apoptosis [15]. Our results indicated that the secreted exendin-4 retains biological activity for enhancement of β-cell insulin secretion and synthesis, which is related to the PDX-1-dependent pathway (Figs 3 and 4). Increased β-cell proliferation and inhibition of β-cell apoptosis were also observed (Fig 5). As displayed in Fig 5, *L*. *paracasei* L14 harboring exendin-4 significantly stimulated cell proliferation and decreased cell apoptosis compared to the strain harboring the empty vector pMG76e. In contrast, when the inhibitor wortmannin was added, there was no significant difference in either cell proliferation or apoptosis following treatment with L14/pMG76e-exendin-4 vs. L14/pMG76e. This proves that the biological activity of recombinant exendin-4 is in fact through the GLP-1 receptor pathway, which comprises the PI3K signaling pathway when exendin-4 is delivered by the *L*. *paracasei* L14 strain. Compared to the blank control (only cell media), *L*. *paracasei* L14 harboring pMG76e and pMG76e-exendin-4 both inhibited cell growth. This could be due to inhibition by *L*. *paracasei* L14 itself. Several studies have reported that probiotic lactic acid bacteria have anticancer effects on cancer cells but have no cytotoxicity toward normal cells [59, 60]. Since INS-1 is a rat insulinoma cell line, inhibition due to *L*. *paracasei*'s anticancer effects is a reasonable assumption. Taken together, our results demonstrate that the engineered strain *L*. *paracasei* L14/pMG76e-exendin-4 can efficiently express and secrete the recombinant exendin-4, and by *in situ* delivery, transform the target protein into biologically active exendin-4, as a GLP-1 receptor agonist.

The Caco-2 cell line, a widely used intestinal model for predicting *in vivo* intestinal drug absorption [61], was used to model for the transport of secreted exendin-4 *in vitro*. The transport rate of exendin-4 delivered by L14/pMG76e-exendin-4 was 34-fold higher than that of the exendin-4 solution from 3–6 h of the experimental period (Fig 6D). Our results are in accordance with previous studies in which the transport rate of GLP-1 was increased by eight times and that of β-lactamase was doubled by *L*. *lactis* delivery [35, 51]. The concentration of exendin-4 on the Caco-2 monolayer may be an important reason for the absorption enhancement. Since the *L*. *paracasei* strain shows strong adhesion to the monolayer, it can secrete exendin-4 directly onto the apical surface of the monolayer and result in a locally high concentration gradient.

This study represents a novel trial for the use of *L*. *paracasei* as a system for oral delivery of exendin-4 for diabetes treatment. As such, it opens up the possibility of oral exendin-4 delivery using live food-grade microorganisms. Many studies have reported that probiotics can control diabetes by improving insulin resistance and alleviating hyperglycemia [62–64]. *L*. *paracasei* L14 was isolated from traditional dairy products, with natural and safe properties, and our previous study also showed that it possesses DPP-IV-inhibitory activity [65] and strong adhesive capacity for intestinal epithelial HT-29 cells, 3.2-fold that of *Lactobacillus rhamnosus* GG. Thus, the recombinant *L*. *paracasei* L14 can colonize the intestinal mucosa and continuously express and deliver the peptide drug *in situ* for long periods. This is desirable and important for diabetes and other chronic diseases. The constitutive p32 promoter used here can maintain the expression of exendin-4 in a cell density-dependent manner without the aid of any expression inducer, such as nisin, after oral administration.

Although the present study shows that *L*. *paracasei* can secrete bioactive exendin-4 and significantly increases the transport of exendin-4 through Caco-2 monolayers, this is only an initial step in exploring the possibility of oral exendin-4 delivery by *L*. *paracasei*. Some issues still need to be addressed before clinical trials, such as the efficacy of *in vivo* delivery, control of the dose of recombinant protein for the desired effect, and potential environmental pollution by the engineered bacteria when eliminated from the body.

## Conclusions

The present work demonstrates that, when transformed by a plasmid, probiotic *L*. *paracasei* can be a live delivery vector for the peptide drug exendin-4. Using lactobacilli as delivery vectors for diabetes treatment opens up the possibility of oral exendin-4 delivery using live food-grade microorganisms. Further investigations into the biological activity of the engineered *Lactobacillus* strain *in vivo* are therefore warranted.

## Supporting Information

S1 Fig The effect of glucose concentration on GLP-1-stimulated insulin secretion from INS-1 cells. Cells were cultured in media with different concentrations of glucose with or without GLP-1 for 2 h, and then insulin concentration was assayed. Medium with 3 mM glucose was used as a control. Data represent the means ± SD of three independent experiments.
(TIF)
