# Supplementary figures and images for: Heterologous Expression and Delivery of Biologically Active Exendin-4 by Lactobacillus paracasei L14
Source: PLoS One. 2016 Oct 20;11(10):e0165130. doi: 10.1371/journal.pone.0165130 (PMC5072737; doi:10.1371/journal.pone.0165130)

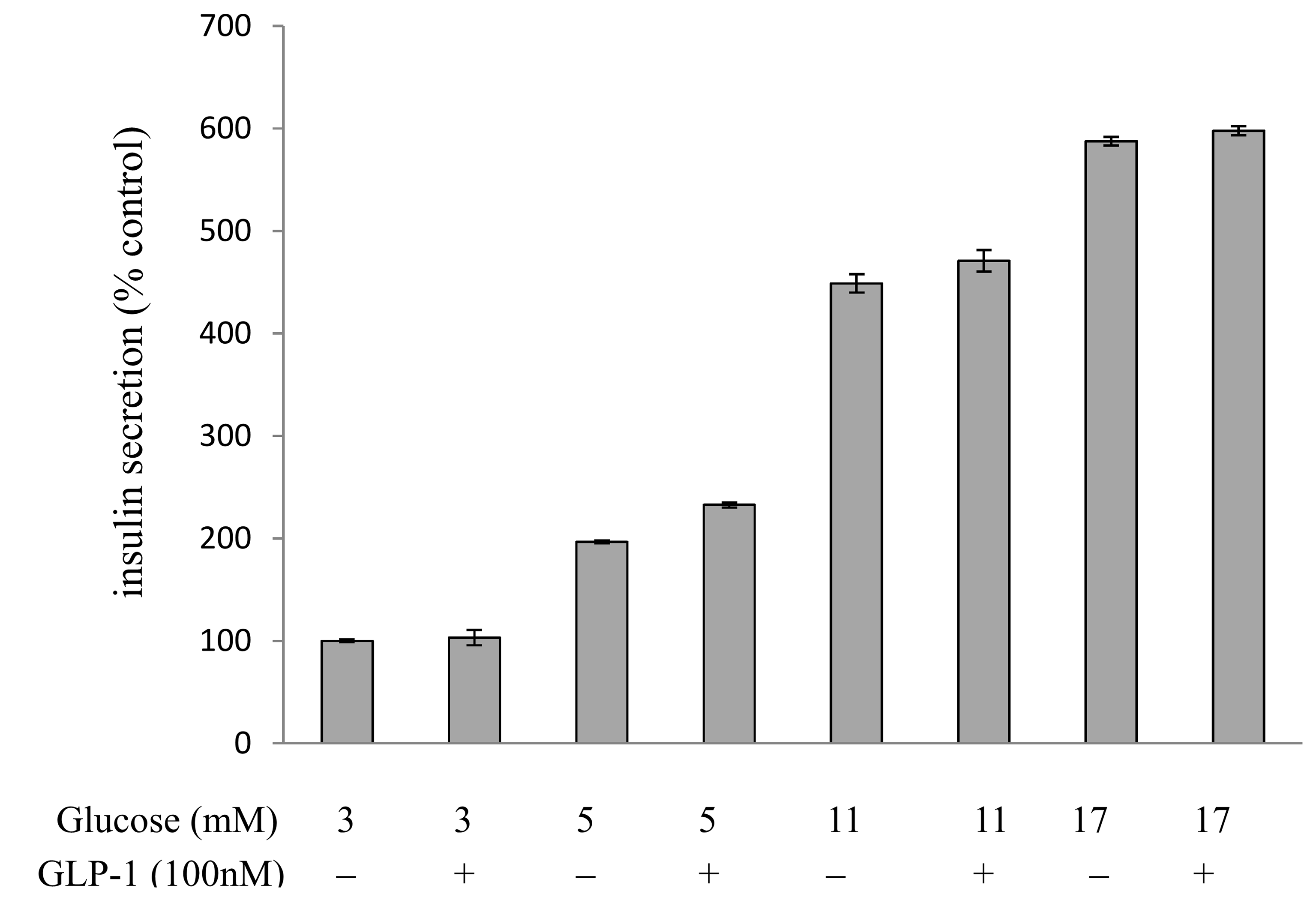

Supplement: S1 Fig — Cells were cultured in media with different concentrations of glucose with or without GLP-1 for 2 h, and then insulin concentration was assayed. Medium with 3 mM glucose was used as a control. Data represent the means ± SD of three independent experiments. (TIF) [file pone.0165130.s001.tif]
